# Cysteamine Supplementation In Vitro Remarkably Promoted Rumen Fermentation Efficiency towards Propionate Production via *Prevotella* Enrichment and Enhancing Antioxidant Capacity

**DOI:** 10.3390/antiox11112233

**Published:** 2022-11-12

**Authors:** Qichao Wu, Hewei Chen, Fan Zhang, Weikang Wang, Fengliang Xiong, Yingyi Liu, Liangkang Lv, Wenjuan Li, Yukun Bo, Hongjian Yang

**Affiliations:** 1 State Key Laboratory of Animal Nutrition, College of Animal Science and Technology, China Agri-Cultural University, Beijing 100193, China; 2 Animal Husbandry Technology Promotion Institution of Zhangjiakou, Zhangjiakou 075000, China

**Keywords:** cysteamine, in vitro fermentation, antioxidant capacity, rumen microbes

## Abstract

Cysteamine (CS) is a vital antioxidant product and nutritional regulator that improves the productive performance of animals. A 2 × 4 factorial in vitro experiment was performed to determine the effect of the CS supplementation levels of 0, 20, 40, and 60 mg/g, based on substrate weight, on the ruminal fermentation, antioxidant capacity, and microorganisms of a high-forage substrate (HF, forage:corn meal = 7:3) in the Statistical Analysis System Institute. After 48 h of incubation, the in vitro dry matter disappearance and gas production in the LF group were higher when compared with a low-forage substrate (LF, forge hay:corn meal = 3:7), which was analyzed via the use of the MIXED procedure of the HF group, and these increased linearly with the increasing CS supplementation (*p* < 0.01). With regard to rumen fermentation, the pH and acetate were lower in the LF group compared to the HF group (*p* < 0.01). However, the ammonia N, microbial crude protein, total volatile fatty acids (VFA), and propionate in the LF group were greater than those in the HF group (*p* < 0.05). With the CS supplementation increasing, the pH, ammonia N, acetate, and A:P decreased linearly, while the microbial crude protein, total VFA, and propionate increased linearly (*p* < 0.01). Greater antioxidant capacity was observed in the LF group, and the increasing CS supplementation linearly increased the superoxide dismutase, catalase, glutathione peroxidase, total antioxidant capacity, glutathione, and glutathione reductase, while it decreased the malondialdehyde (*p* < 0.05). No difference occurred in the ruminal bacteria alpha diversity with the increasing CS supplementation, but it was higher in the LF group than in the HF group (*p* < 0.01). Based on the rumen bacterial community, a higher proportion of *Bacteroidota*, instead of *Firmicutes*, was in the LF group than in the HF group. Furthermore, increasing the CS supplementation linearly increased the relative abundance of *Prevotella*, *norank_f_F082*, and *Prevotellaceae_UCG-001* under the two substrates (*p* < 0.05). *Prevotella*, *norank_f_F082*, and *Prevotellaceae_UCG-001* were positively correlated with gas production, rumen fermentation, and antioxidant capacity in a Spearman correlation analysis (r > 0.31, *p* < 0.05). Overall, a CS supplementation of not less than 20 mg/g based on substrate weight enhanced the rumen fermentation and rumen antioxidant capacity of the fermentation system, and it guided the rumen fermentation towards glucogenic propionate by enriching the *Prevotella* in *Bacteroidetes*.

## 1. Introduction

The rumen converts various ingested feed components into proteins, volatile fatty acids, and vitamins via the diverse and complex microbial ecosystem of ruminants [1]. The improvement of rumen fermentation efficiency depends on a stable and healthy rumen environment [2], which generally leads to the improvement of animal performance [3]. The health and biological functioning of livestock are often prioritized [4,5]. The development of feed additives that can improve animal performance and that consider animal health is one of the hot fields worldwide, especially when adding some beneficial additives to feed may affect animal production and health [6,7], as well as enhance productivity in livestock [8,9].

Reactive oxygen species (ROS) in animals are vital electron acceptors with a double free radical structure, including superoxide (O^2−^), hydrogen peroxide (H_2_O_2_), and other free radicals [10]. ROS strongly attack the nucleic acids, proteins, and lipids in the cells of animals, and they chemically destroy the organic components of cells [11,12]. ROS production is generally related to oxidative stress and oxidative metabolism. Oxidative stress in ruminants was reported to reduce animal performance, such as in lower growth performance and lower milk yield [13].

As an amino thiol, cysteamine (CS; β-Mercaptoethylamine) is an amino thiol derived from coenzyme A degradation, and it is closely related to the production of cysteine and glutathione (GSH). In previous studies, CS has been shown to inactivate somatostatin and thus increase growth hormone concentrations in animals [14,15,16]. According to a study, a dietary CS supplementation of less than 60 mg/kg BW increased the growth rate and improved the feed efficiency to varying degrees in feedlot lambs [17]. In another study, 30 and 45 g/day of dietary CS supplementation increased the milk yield and milk protein content in lactating cows [18]. However, limited data were obtained about the effect of CS supplementation on rumen fermentation and microorganisms. In addition, as a reservoir for cysteine, GSH is the major non-protein sulfydryl compound in mammalian cells, performing an essential role in protecting the cell from oxidative damage [19]. Based on previous studies, the supplementation of CS to the in vitro maturation increases intracellular glutathione synthesis in bovines [20]. In particular, higher superoxide dismutase (SOD) and glutathione peroxidase (GPx) antioxidant activities were achieved with the addition of 2.5–7.5 mM of CS to post-thaw semen [21]. The greater SOD activity and the mRNA expression of interleukin-10 in serum occurred in weaned pigs fed 80 mg/kg CS [22]. To sum up, CS exhibits antioxidant properties under some conditions. However, related studies on the effect of CS supplementation on the antioxidant capacity of the rumen environment have not been reported.

Although CS as a feed additive has been widely reported with regard to the growth performance of animals, the studies on rumen fermentation, antioxidant capacity and rumen microorganisms are relatively lacking. Therefore, the present study investigated the effects of different concentrations of CS on rumen fermentation efficiency, the antioxidant capacity of the rumen environment, and the rumen microflora under the in vitro culture and fermentation conditions of two different substrates.

## 2. Materials and Methods

### 2.1. Preparation of CS Products

The CS products were purchased commercially from Sigma Aldrich (St. Louis, MO, USA) and contained 900 g/kg of dry matter, less than 2 mg/kg of Pb, and less than 2 mg/kg of As. To prevent oxidation in the air, the CS product was supplied in the form of hydrochloride and stored at 4 °C in a refrigerator prior to the experiment.

### 2.2. In Vitro Batch Cultures

As the forage samples, *L. chinensis* hay was harvested immediately at the early bloom stage and chopped into 35 mm pieces. Then, it was oven dried at 65 °C for 48 h, and the forage samples were ground in a Wiley mill to pass through a 2.0 mm sieve for the subsequent experiment. Corn meal was purchased from the local feed market. The forage-to-concentrate ratio was mixed at 7:3 and 3:7 as two substrates in the in vitro batch culture experiments. The nutritional composition of the two substrates is shown in Table 1.

Five rumen-cannulated Dorper × Hu hybrid male sheep at seven months of age with an initial live body weight of 38.32 ± 0.94 kg served as donor animals for the collection of the rumen fluids. The sheep were housed in the same pens with a fecal leaking floor. The fresh water was provided ad libitum and each sheep was fed 500 g foxtail millet silage, 500 g cornstalk, 650 g corn meal, 300 g soybean meal, and 50 g premix daily. Through rumen fistulas, rumen fluid from each sheep was collected 3 h after morning feeding from different sites inside the rumen and squeezed through four layers of medical-use cheesecloth. Then, the rumen fluid samples were mixed in equal proportions and immediately stored in pre-heated vacuum bottles surrounded by carbon dioxide to maintain an anaerobic environment. The Animal Ethics Committee of China Agricultural University approved all the procedures with animals. The sampling procedures followed the Guidelines on Ethical Treatment of Experimental Animals (2006) No. 398 set by the Ministry of Science and Technology, Beijing, China.

A completely random design was conducted for the in vitro culture (2 substrates × 4 concentrations × 7 replicates). The 56 glass bottles with a volume of 120 mL were divided into two substrate treatments (LF, forage:corn meal = 3:7; HF, forage:corn meal = 7:3) and four different CS supplementation concentrations (0, 20, 40, and 60 mg/g based on substrate weight). The 0.5 g substrate, 50 mL buffer of preheated culture medium at 39 °C [22], and 25 mL filtered rumen fluid were added into each bottle. The batch cultures were carried out in the Automated Trace Gas Recording System for Microbial Fermentation (AGRS) at 39 °C. Cumulative gas production (GP) was recorded continuously by connecting the glass bottles to the gas inlets of the equipment and incubating continuously for 48 h in the experiment. After 48 h of incubation, in vitro dry matter disappearance at 48 h (IVDMD_48_) was determined by filtering the content of each bottle through a nylon bag (8 × 12 cm, 42 µm pore size). Meanwhile, the pH was determined using a portable pH meter (PHS-2F, INESA Scientific Instrument, Shanghai, China). Then, 1 mL filtered culture fluid was sampled into Dnase-free polypropylene tubes (stored at −80 °C) for the volatile fatty acid (VFA), ammonia-N (NH_3_-N), microbial protein (MCP), antioxidant capacity, and bacterial community analysis (*n* = 6). At the end of the in vitro fermentation experiment, all samples were collected immediately, and the relevant indexes were determined within 3 days after the experiment, but the gene sequencing and analysis lasted for one month.

### 2.3. Determination of In Vitro Dry Matter Digestibility, Volatile Fatty Acids, and Antioxidant Content

IVDMD_48_ was calculated based on the difference between the dry matter (DM) weight of the substrate before incubating and the residual content in the nylon bags (*n*= 7). Based on the method described by Chaney and Marbach [23], the NH_3_-N concentrations were measured by using a microplate reader (RT-6500, Rayto, Shanghai, China). The concentrations of MCP were determined following the Bradford and Williams method [24]. Then, 1 mL of the culture fluid samples was added to 300 μL metaphosphoric acid (25%, *w*/*v*) and placed at 4 °C for 30 min. After centrifugation at 11,000× *g* for 20 min, the VFA was determined by using gas chromatography (GC522, Wufeng, Shanghai, China) to inject the supernatant samples. The superoxide dismutase (SOD), total antioxidant capacity (T-AOC), malondialdehyde (MDA), glutathione peroxidase (GSH-Px), glutathione (GSH), glutathione reductase (GR), and catalase (CAT) were determined according to the manufacturer’s instructions for the enzyme-linked immunosorbent assay (ELISA) kits (KL-GH-S, Conlon Biotechnology Co., Shanghai, China).

### 2.4. DNA Extraction and Sequencing

Total DNA was extracted from the rumen fluid samples according to the instructions of an E.Z.N.A.^®^ soil DNA kit (Omega Bio-Tek, Norcross, GA, USA). The quality of the DNA extraction was determined using 1% agarose gel electrophoresis, and the DNA concentration and purity were determined using a NanoDrop2000. The hypervariable region V3-V4 of the bacterial 16S rRNA gene was amplified with 338F (5′-ACTCCTACGG-GAGGCAGCAG-3′) and 806R (5′GGACTACHVGGGTWTCTAAT-3′) by an ABI GeneAmp^®^ 9700 PCR thermocycler (ABI, Los Angeles, CA, USA). The amplification procedure was as follows: initial denaturation at 95 °C for 3 min, followed by 27 cycles of denaturing at 95 °C for 30 s, annealing at 55 °C for 30 s, and extension at 72 °C for 45 s, with a single extension at 72 °C for 10 min, ending at 4 °C. The PCR mixtures contained 5× TransStart FastPfu buffer 4 μL, 2.5 mM dNTPs 2 μL, forward primer (5 μM) 0.8 μL, reverse primer (5 μM) 0.8 μL, TransStart FastPfu DNA Polymerase 0.4 μL, template DNA 10 ng, and finally ddH2O up to 20 μL. PCR reactions were performed in triplicate. The PCR product was extracted from 2% agarose gel and purified using the AxyPrep DNA Gel Extraction Kit (Axygen Biosciences, Union City, CA, USA) according to the manufacturer’s instructions and quantified using a Quantus™ Fluorometer (Promega, Madison, WI, USA). Purified amplicons were pooled in equimolar amounts and paired-end sequenced on an Illu-mina MiSeq PE300 platform/NovaSeq PE250 platform (Illumina, San Diego, CA, USA) according to the standard protocols of Majorbio Bio-Pharm Technology Co., Ltd. (Shanghai, China). The raw reads were deposited into the NCBI Sequence Read Archive (SRA) database (Accession Number: PRJNA877565).

The raw 16S rRNA gene sequencing reads were demultiplexed, quality-filtered by FAST version 0.20.0, and merged by FLASH version 1.2.7 [25]. Operational taxonomic units (OTUs) with a 97% similarity cutoff [26,27] were clustered using UPARSE version 7.1 [26], and the chimeric sequences were identified and removed. The taxonomy of each OUT representative sequence was analyzed using the RDP Classifier version 2.2 [28] against the 16S rRNA database (e.g., Silva v138), using a confidence threshold of 0.7.

### 2.5. Chemical Analyses

Based on the Association of Official Analytical Chemists [29], the ash, crude protein, and ether extract of the substrate samples were analyzed. In addition, the neutral detergent fiber (NDF) and acid detergent fiber (ADF) were determined with the approach employed by Van Soest et al. [30].

### 2.6. Calculations

The real-time gas production data recorded by the automatic gas production recording device were imported into SAS 9.4 and fitted with the non-linear (NLIN) procedure of SAS 9.4 (Statistical Analysis for Windows, SAS Institute Inc., Cary, NC, USA) based on the France et al. [31] model using Equation (1):

GP_t_ = A/[1 + (C/t) B] (1)

where GPt is the cumulative gas production at time t (h); A is the estimated asymptotic gas production (mL/g DM); t is the time of the gas recording; B is a sharpness parameter determining the shape of the curve, and C is the time (h) at which half of A is reached.

The average gas production rate (AGPR, mL/h) was calculated according to the Wang et al. [32] model via the use of Equation (2):

AGPR = (A × B)/(4 × C)(2)

### 2.7. Statistical Analysis

The data passed the normality test before the parametric analysis application in the present study. The data of each substrate (LF and HF) were analyzed using the MIXED procedure of the Statistical Analysis System Institute (SAS, 2003). The model was applied as follows:

Yijk = μ + Gi + Fj + (G × F)ij + eijk(3)

where Yijk is the dependent variable, µ is the overall mean, Gi is the fixed effect of increasing the CS concentration (i = 4:0, 20, 40, and 60 mg/g based on substrate weight), Fj is the fixed effect of the substrate type with different forage:corn meal (7:3 and 3:7), G × F is the interaction of the substrate type and CS concentration. Eijk is the residue error term. The least square means and standard errors of the means were calculated with the LSMEANS statement of the SAS software. Significance was declared at *p* < 0.05 unless otherwise noted.

## 3. Results

### 3.1. In Vitro Dry Matter Disappearance at 48 h and Kinetic Gas Production

**Figure 1:**
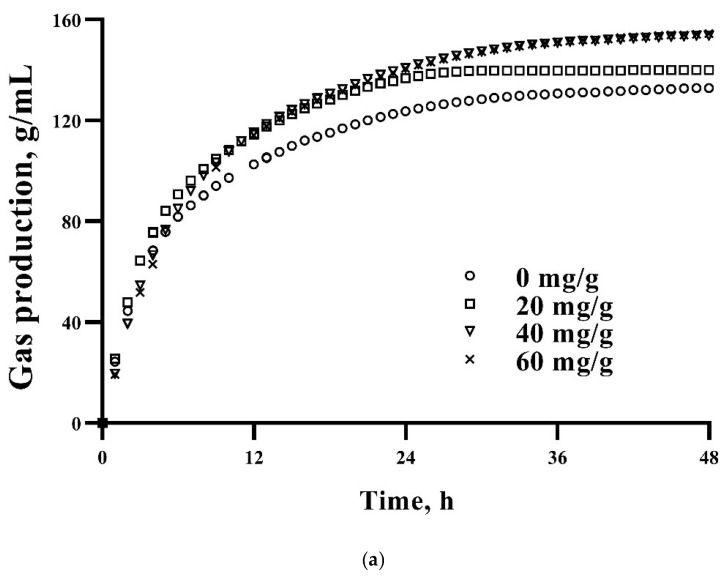

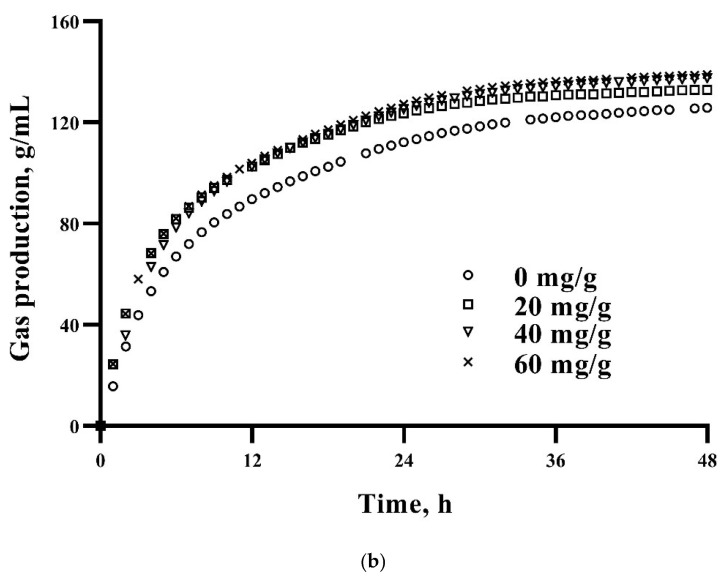
Cumulative gas production profiles of in vitro fermentation of substrates with LF (**a**) and HF (**b**) in response to increasing CS supplementation in culture fluids at 48 h.

The gas production kinetic parameters of the culture fluids are shown in Table 2. The IVDMD_48_ in the LF group was significantly greater than that of the HF group (*p* < 0.01) and increasing the CS supplementation level linearly increased the IVDMD_48_ (*p* = 0.04). With regard to the kinetic parameters, the GP_48_, A, B, and AGPR in the LF group were significantly higher than those of the HF group (*p* < 0.01), and a linear increase was observed in GP48 with the increasing CS supplementation (*p* < 0.01). Furthermore, increasing the CS supplementation linearly increased *A*, *B*, and *AGPR* (*p* < 0.01). However, *C* in the LF group was significantly lower than that of the HF group (*p* < 0.01) and increasing the CS supplementation quadratically decreased *C* (*p* < 0.01). The interaction effect occurred in GP_48_ and *AGPR*. As Figure 1 shows, the maximum gas production in the LF group (a) was higher than that of the HF group (b) and increasing the CS supplementation linearly increased the gas production (*p* < 0.01).

### 3.2. Rumen Fermentation

As shown in Table 3, the pH in the LF group was significantly lower than that of the HF group, while the ammonia N, MCP, and total VFA in the LF group were higher compared with those of the HF group (*p* < 0.01). In line with CS0, the groups with CS added were lower in pH, though the linear and quadratic effect was not significant. Furthermore, increasing the CS supplementation linearly increased the MCP and total VFA but decreased the ammonia N (*p* < 0.01). The interaction effect only occurred in the MCP. With regard to the VFA composition, the acetate in the LF group was lower than that of the HF group, but the opposing situation was observed in the propionate (*p* < 0.05). As the CS supplementation increased, it linearly decreased the acetate but quadratically increased the propionate (*p* < 0.05). As a result, a quadratic decrease occurred in A:P under the two substrates (*p* < 0.01). The interaction effect was observed in the propionate. However, the substrates and the increasing CS supplementation did not affect the butyrate.

### 3.3. Rumen Antioxidant Capacity

The effect of the antioxidant capacity is shown in Table 4. Compared with the HF group, the SOD, GSH-Px, T-AOC, and GR in the LF group were higher, while the MDA in the LF was lower (*p* < 0.01). However, the type of substrate did not affect the CAT and GSH. The increasing CS supplementation in the fermentation system linearly increased the SOD, CAT, GAS-Px, T-AOC, GSH, and GR, while the MDA decreased linearly with the increasing CS supplementation (*p* < 0.05). Moreover, the interaction effect occurred in the CAT, GSH-Px, T-AOC, GSH, and GR (*p* < 0.05) instead of the SOD and MDA.

### 3.4. Rumen Bacteria Community

As shown in Table 5, the coverage was 0.99 in all the samples with the different CS supplementation under the two substrates. Furthermore, the index of alpha diversity was closely related to the type of substrate. Based on the result, the indexes in the LF group, such as Chao, Ace, Shannon, and Sobs (*p* < 0.01), were significantly higher than those of the HF group. However, Simpson was lower in the LF group compared with the HF group (*p* < 0.01). Then, the type of substrate in the fermentation system did not affect the ruminal bacteria alpha diversity based on the OTUs. The interaction effect between the substrates and the CS supplementation level was not observed in the present study.

**Figure 2:**
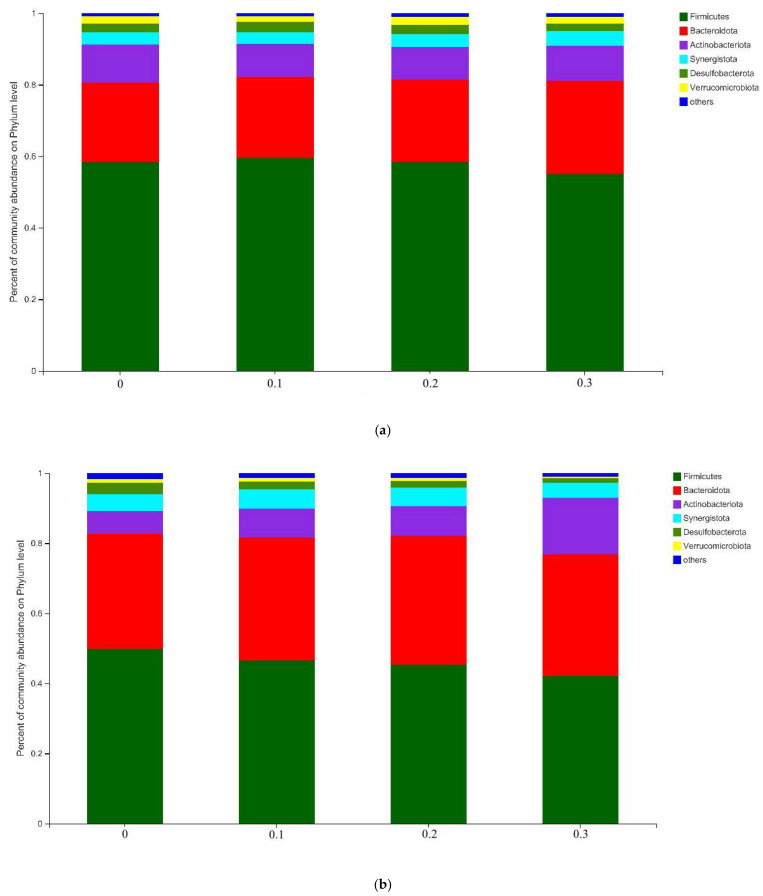
Bacterial community of fermentation fluids supplementing increasing CS level based on the phylum level in HF group (**a**) and LF group (**b**).

In the microbial community based on the phylum level (Figure 2), *Firmicutes*, *Bacteroidota*, *Acitinobacteriota*, *Synergistota*, *Desulfobacterota*, and *Verrucomicrobiota* were determined as the predominant phyla with a relative abundance of >1% in the present study. Furthermore, *Firmicutes* (42.2–49.8%) and *Bacteroidota* (32.9–36.9%) were the largest bacterial phyla in the LF group, together accounting for 86.7% of all the bacteria. The remaining phyla in the LF group accounted for 6.5–16% of *Acitinobacteriota*, 4.4–5.6% of *Synergistota*, 1.3–3.3% of *Desulfobacterota*, and 0.8–1.6% of *Verrucomicrobiota* in the LF group. However, compared with the LF group, the relative abundance of *Firmicutes* (55.0–59.5%) was higher, while the relative abundance of *Bacteroidota* (22.2–26.1%) was lower (*p* < 0.01). The remaining phylum taxonomic compositions in the HF group were *Acitinobacteriota* (9.2–10.2%), *Synergistota* (3.3–4.0%), *Desulfobacterota* (2.1–2.9%), and *Verrucomicrobiota* (1.5–2.2%). Increasing the CS supplementation did not change the predominant phyla in the fermentation environment under the two substrates in the present study.

At the genus level, 18 genera were determined as the predominant phyla with a relative abundance of >1% in the present study. Among these predominant genera, the highest relative abundance occurred in *Succiniclasticum* (18.9–21.2%) in the HF group and *Rikenellaceae_RC9_gut* (12.2–13.5%) in the LF group (Table 6 and Table 7). Under the two substrates, the genera that differed in composition were mainly those with low relative abundance, including *norank_f_Muribaculaceae* (6.36–7.86%), *norank_o_WCHB1-41* (1.19–1.93%), *Eubacterium_nodatum* (1.45–1.62%), and *Veillonellaceae_UCG-001* (0.99–1.32%) in the HF group and *Muribaculaceae* (5.85–8.33%), *Prevotellaceae_UCG-003* (0.75–1.14%), and *Sharpea* (0.59–1.71%) in the LF group (Table 6 and Table 7). Furthermore, increasing the CS supplementation linearly increased *Prevotella*, *norank_f_F082*, and *Prevotellaceae_UCG-001* but decreased *norank_f_Eubacterium_coprostanoligenes* and *Family_XIII_AD3011* in the HF group (*p* < 0.05). In the LF group, there a linear increase was observed in *Bifidobacterium*, *Prevotella*, *norank_f_F082*, *Prevotellaceae_UCG-001*, and *Sharpea*, while a linear decrease occurred in *Christensenellaceae_R-7*, *Desulfovibrio*, *Ruminococcus*, and *Family_XIII_AD3011* in the LF group (*p* < 0.01). Additionally, no significant difference was observed in the other genera among the four treatments under the two substrates.

### 3.5. Correlations among the Top 10 Bacterial Genera and the Parameters of Gas Production Kinetic Parameters, Rumen Fermentation, and Antioxidant Capacity

**Figure 3:**
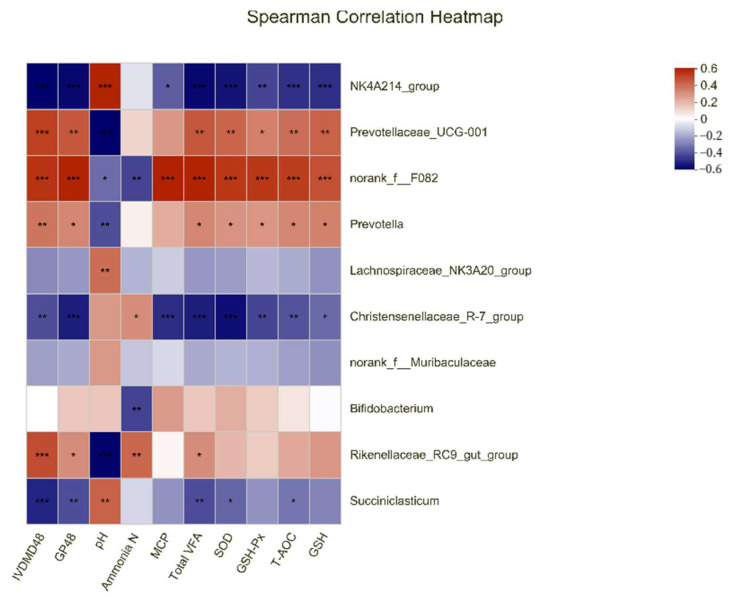
Heat map of the correlations among the top 10 bacterial genera and the parameters of gas production, rumen fermentation, and antioxidant capacity. Color intensity represents *p*−values of correlation, * *p* ≤ 0.05, ** *p* ≤ 0.01, *** *p* ≤ 0.001.

According to the heat map analysis shown in Figure 3, the IVDMD_48_ and GP_48_ were negatively correlated with the presence of *NK4A214_group*, *Christensenellaceae_R-7*, and *Succiniclasticum* (r < −0.39, *p* < 0.01), while they were positively correlated with *Prevotellaceae_UCG-001*, *norank_f_F082*, *Prevotella*, and *Rikenellaceae_RC9_gut* (r > 0.31, *p* < 0.05). The pH was positively related to *NK4A214_group*, *Lachnospiraceae_NK3A20*, and *Succiniclasticum* (r > 0.39, *p* < 0.01), but it was negatively correlated with *Prevotellaceae_UCG-001*, *norank_f_F082*, *Prevotella*, and *Rikenellaceae_RC9_gut* (r < −0.33, *p* < 0.05). In the nitrogen metabolism of rumen, ammonia N was only negatively correlated with *norank_f_F082* and *Bifidobacterium* (r < −0.42, *p* < 0.01) and positively related to *Christensenellaceae_R-7* and *Rikenellaceae_RC9_gut* (r > 0.31, *p* < 0.05). However, the MCP was positively related to *norank_f_F082* (r > 0.59, *p* < 0.01) and negatively correlated with *NK4A214_group* and *Christensenellaceae_R-7* (r < −0.36, *p* < 0.05). Furthermore, the total VFA, SOD, GSH-Px, T-AOC, and GSH were negatively correlated with the presence of *NK4A214_group* and *Christensenellaceae_R-7* (r < −0.35, *p* < 0.05) and positively correlated with *Prevotellaceae_UCG-001*, *norank_f_F082*, and *Prevotella* (r > 0.28, *p* < 0.05). Only the total VFA, SOD, and T-AOC were negatively correlated with *Succiniclasticum* (r < −0.34, *p* < 0.05).

## 4. Discussion

The technology of in vitro simulated rumen fermentation can be used to evaluate the digestibility of the feed, the related indexes of rumen fermentation, and the changes in the rumen environment [33]. This technology is complemented by AGRS technology developed in the laboratory to evaluate the real-time efficiency of rumen fermentation based on real-time gas production. In the present study, this method was used to explore the kinetic gas production, rumen fermentation, rumen antioxidant capacity, and rumen bacteria community.

Antioxidants generally scavenge free radicals by enhancing the activity of antioxidant enzymes. SOD, for example, catalyzes the reaction between superoxide free radicals and hydrogen ions to remove free radicals with oxidative damage in the cells, during which a large amount of hydrogen ions are consumed [34]. In the rumen, the hydrogen ions are catalyzed by antioxidant enzymes to scavenge free radicals, resulting in a decrease in the partial pressure of the hydrogen in the rumen. According to the relevant studies, the lower partial pressure of hydrogen in the rumen contributes to the rumen fermentation and the production of propionate in the rumen [35]. Therefore, the present study speculated that the enhancement of the antioxidant capacity in the rumen environment is more conducive to propionate fermentation.

In general, the gas production extent and rate are the vital indexes for determining kinetic rumen fermentation in in vitro simulated rumen fermentation. Based on the study of Kumar, decreasing roughage ratios (47.67, 61.67, and 67.33% for the ratios 80:20, 50:50, and 20:80, respectively) increased the IVDMD in rumen fermentation [36]; this was the same as the result in the present study. Previous studies in vitro found that low-forage substrates (hay:concentrate = 1:4) promoted rumen fermentation efficiency instead of high-forage (hay:concentrate = 4:1), including higher IVDMD_48_, gas production extent, and rate [37]. In rumen fermentation, the high-forage fermentation substrate generally led to an increase in the number of fiber-degrading microorganisms, while the low-forage fermentation substrate resulted in an increase in the number of starch-degrading microorganisms. The soluble and easily degraded components in feeds (e.g., starch) are always utilized by the rumen microbes first [38]; the gas production extent and the rate of low-forage fermentation substrate were higher than those of the high-forage fermentation substrate, which was also one of the reasons why flatulence and acidosis were easily caused in ruminants feeding on a low-forage diet. In the present study, increasing the CS supplementation also linearly increased the IVDMD_48_ and gas production, which might be explained by the enhancement of the starch degradation. According to the previous experimental findings, a CS addition higher than 20 mg/kg BW to the diet of fattening lambs significantly increased the abundance of *Prevotella* with a linear dose effect [17]. The main role of *Prevotella* was to promote starch degradation in the rumen [39], which led to an increase in the extent and rate of gas production in the rumen. This result was consistent with the in vitro simulation of rumen fermentation in the present experiment, although different from the experimental CS addition concentration. In sum, the low-forage substrate and increasing CS supplementation promoted substrate digestibility, gas production, and fermentation rate in rumen.

To remain as a stable ruminal environment, an optimal pH is vital and essential [40]. In the present study, the low-forage substrate resulted in a lower pH, which could be due to the degradation characteristics of the substrate components. NH_3_-N is the metabolic product of diet protein metabolism and the precursor of microbial protein synthesis in rumen. According to a previous study, more than 100 mg/L CS in in vitro fermentation decreased the production of NH_3_-N and increased MCP [41]. As a pivotal role in various metabolisms, VFAs are the main energy source (75% of their digestible energy) of ruminants [42]. In the present study, a significantly higher total VFA in the LF group than in the HF group was explained by the more easily degraded properties of the substrate in fermentation. A study on weaned lambs with fistulated rumen suggested that 150 mg/kg BW of CS supplementation in the diet significantly elevated the lactate dehydrogenase activity and total VFA [43], which indicated the promoting effect in rumen fermentation with the adding of CS. In the present study, increasing the CS supplementation indeed enhanced the rumen fermentation efficiency, as is reflected in the linear increase in the total VFA concentration. Furthermore, the same results were observed in an in vitro fermentation study [41], in which higher VFA production occurred in the fermentation fluid incubated with CS in the experiment. Because of the nature of the substrate (mainly structural carbohydrates), the bacteria easily converted the carbohydrate into acetate instead of propionate [44]. In the present study, the higher acetate and lower propionate in the HF group also supported this point. In the present study, increasing the CS supplementation strengthened the production of the propionate instead of the acetate, which led to a linear decrease in A:P. An earlier study found that the basal rations addition of CS in Yaks (5 g/day each yak, 30% purity) significantly promoted the propionate compared to the control group [45]. CS was reported to increase the propionate in the fluids incubated with 50 mg/L of CS in the in vitro rumen fermentation of goats [41]. As the primary precursor of gluconeogenesis in the rumen, propionate made a significant net contribution to their glucose synthesis to gain more energy supply for the animals in order to obtain a higher production performance. According to the subsequent analysis of the results, the enrichment effect of CS on Prevotella could explain the promotion effect of CS on rumen fermentation and the transformation of the rumen fermentation type. In addition, the enhanced antioxidant capacity of the rumen environment may also explain the promoting effect in the rumen fermentation and propionic acid fermentation by the depletion of the rumen hydrogen partial pressure.

In sum, increasing the CS supplementation not less than 20 mg/g based on the substrate weight promoted the rumen fermentation efficiency and changed the rumen fermentation pattern towards glucogenic propionate.

In ruminants, the weaning and high-concentrate feeding of dairy cows or fattening sheep cause loss of balance in the rumen environment and produce oxidative stress [46,47,48]. In animal husbandry, how to enhance the antioxidant capacity of animals has attracted more and more attention. SOD is involved in the free radical scavenging process in the body cells [49]. The GSH-Px, CAT, and T-SOD are the most vital antioxidant enzymes to eliminate unwanted lipid hydroperoxide and hydrogen peroxide [50]. MDA is the oxidation end product of hydrogen peroxide and is an indicator of oxidative damage in DNA, lipids, and proteins [51]. In the present study, increasing the CS supplementation significantly increased the SOD, CAT, GSH-Px, T-AOC, GSH, and GR but decreased the MDA, suggesting that CS indeed enhanced the antioxidant capacity in rumen fermentation. There was a linear correlation between the increased rumen antioxidant capacity and the CS supplementation level in the present study. There are limited studies on the antioxidant effects of CS using an in vitro rumen fermentation experiment. A related study showed that 20 and 50 mg/kg of BW CS had a positive effect on the secretion of the antioxidant status in the growing lambs grazed on mountain pasture [52], which was consistent with the enhanced antioxidant capacity caused by adding CS in the present study. In pigs, the greater SOD and the expression of interleukin-10 (IL-10) mRNA were observed in weaned pigs fed a diet with the addition of 80 mg/kg BW CS [21]. Furthermore, based on a study exploring the antioxidant effect of CS on semen preservation, the addition of 2.5 and 7.5 mM of CS to the semen extender provided a higher increase in SOD and GPx antioxidant enzyme activities [53]. The present study suspected that the antioxidant ability of CS might be related to its thiol group, which could serve as an antioxidant in situ [54]. Secondly, CS was reported to react with cysteine through a thioalkyl-disulfide exchange to form mixed disulfide [55], which could enhance the synthesis of glutathione to scavenge free radicals in the body. Glutathione is a potent intracellular antioxidant that influences cellular redox homeostasis [56]. Therefore, CS could be used as a new type of antioxidant in the practical production of ruminants. According to the viewpoints discussed earlier, the increased antioxidant capacity of the rumen might be beneficial in promoting the rumen fermentation and the shift to propionic acid fermentation.

In sum, increasing the CS supplementation strengthened the antioxidant capacity in rumen fermentation, which might be explained by the presence of its thiol group and the promotion of glutathione synthesis.

Rumen microorganisms play an enormous role in ruminant productivity. Involved in the degradation of plant carbohydrates and their subsequent conversion to short-chain fatty acids [57], rumen bacteria provide energy for the basic metabolic processes and also play an irreplaceable role in the fatty acid metabolism of dietary fat [58]. In the present study, the results on the ruminal bacteria alpha diversity based on the OTUs suggested that CS supplementation did not affect the diversity of microorganisms, but the LF substrate increased the diversity compared to the HF substrate. This indicated that the type of fermentation substrate could indeed affect the diversity of the rumen microorganisms. *Firmicutes*, *Bacteroidota*, and *Acitinobacteriota* were determined as the dominant phyla in rumen fermentation, which was consistent with the study in vivo [17,59]. At the genus level, it was worth noting that *Prevotella*, *norank_f_F082*, and *Prevotellaceae_UCG-001* increased linearly with the addition of the increasing CS level under the two fermentation substrates in the present study. *Prevotella* and *Prevotellaceae_UCG-001* belong to the family of *Prevotellaceae*. As the core genus of *Bacteroidetes*, *Prevotella* performed a vital role in secreting enzymes, degrading the starch to produce propionate or propionate-precursor succinate [60], which might restrict fiber fermentation in the rumen [61]. In the rumen, the non-structural carbohydrates (such as starch) were rapidly and efficiently degraded to glucose with the presence of *Prevotella*; then, large amounts of pyruvate were produced via the glycolytic pathway and were the major precursor of VFA in the rumen [62,63]. Earlier studies found that the presence of *Prevotella* promoted the production of propionate through gluconeogenesis in the rumen, which was used to obtain better growth performance and feed conversion efficiency in ruminants [64], and also explained the positive correlation between *Prevotella* and IVDMD_48_, GP_48_, MCP, total VFA, and propionate in the present study. On the other hand, *Prevotella* was also reported to be involved in the degradation of peptides into amino acids [65]. There were restricted reports on *norank_f_F082* in ruminants. Both *norank_f_F082* and *Prevotella* belong to *Bacteroidetes*, which was mainly involved in the degradation of non-structural carbohydrates. The linear increase in *Bifidobacterium* in the LF group was also noteworthy in the present study. As bacteria producing carbohydrate-degrading enzymes, *Bifidobacterium* promoted the metabolism of various dietary carbohydrates, which assisted the host to absorb energy and improve feed efficiency [66]. Moreover, *NK4A214_group*, *Christensenellaceae_R-7*, and *Succiniclasticum* all belonged to the phylum of *Firmicutes*, which mainly participated in the degradation of cellulose and hemicellulose, resulting in acetate fermentation in the rumen [67]. Acetate fermentation is generally slow and provides a lower energy supply [68], which might be explained by the negative correlation between these bacteria and IVDMD_48_, GP_48_, MCP, and total VFA. Due to its own characteristics in autioxidation, CS supplementation enhanced the antioxidant capacity, and meanwhile, it changed the metabolism in rumen by increasing the related bacteria, especially *Prevotella*. Based on these, the positive correlation between the antioxidant properties and *Prevotella*, *norank_f_F082*, and *Prevotellaceae_UCG-001* could be explained.

Taken together, the results on the rumen bacteria community suggested that CS supplementation guided rumen fermentation towards a glucogenic propionate by enriching *Prevotella* in *Bacteroidetes*.

## 5. Conclusions

The present study investigated the effects of different concentrations of CS on rumen fermentation efficiency, the antioxidant capacity of the rumen environment, and the rumen microflora under the in vitro culture and fermentation conditions of the two different substrates. The CS supplementation of not less than 20 mg/g significantly increased the in vitro rumen gas production extent and rate regardless of whether low- or high-forage diets were fermented. Meanwhile, the CS supplementation promoted the growth of rumen microbes via the remarkable utilization of the ammonia N and increased VFA production. Interestingly, the present study observed for the first time that the antioxidant capacity in the fermentation system was significantly enhanced, as indicated by the SOD, etc., likely via consuming the hydrogen yielded in the rumen fermentation. Furthermore, the CS supplementation shifted the rumen fermentation towards glucogenic propionate production by enriching the *Prevotella* in *Bacteroidetes*.

## Figures and Tables

**Table 1:** Composition and nutrient concentrations of fermentation substrates in experiment (g/kg DM).

Items ^1^	Low-Forage (LF)	High-Forage (HF)
Fermentation substrates composition		
*L. chinensis* hay	300	700
Corn meal	700	300
Nutrition concentrations		
CP	81.2	73.9
EE	26.3	16.3
NDF	153.1	547.2
ADF	294.0	323.8
NFC	727.3	350.2
Ash	12.1	12.4

^1^ CP, crude protein; EE, ether extract; NDF, neutral detergent fiber; ADF, acid detergent fiber; NFC, non-fiber carbohydrate; NFC = 1000 − (NDF g/kg DM + CP g/kg DM + EE g/kg DM + Ash g/kg DM).

**Table 2:** In vitro dry matter disappearance and gas production kinetic parameters at 48 h of culture fluids under two substrates and different CS supplementation level.

Item ^1^		CS Supplementation Level (mg/g Based on Substrate Weight)				SEM	*p*-Value ^2^			
		0	20	40	60		S	I	L	Q
IVDMD_48_, g/kg DM	LF	71.8	73.1	75.9	77.7	2.34	<0.01	0.16	0.04	0.42
	HF	56.9	59.0	62.2	61.5					
GP_48_, mL/g DM	LF	133.7	139.8	152.5	154.5	2.06	<0.01	0.038	<0.01	0.319
	HF	126.9	131.5	137.6	138.1					
*A*, mL/g DM	LF	148.7	156.4	165.6	167.5	2.31	<0.01	0.441	<0.01	0.267
	HF	136.3	140.8	151.4	151.8					
*B*	LF	1.05	1.21	1.37	1.35	0.023	<0.01	0.121	<0.01	<0.01
	HF	0.96	1.07	1.20	1.21					
*C*, h	LF	6.40	5.43	3.93	3.87	0.094	<0.01	0.457	<0.01	<0.01
	HF	6.83	5.64	4.36	4.40					
*AGPR*, mL/h	LF	6.11	8.75	14.49	14.66	0.503	<0.01	<0.01	<0.01	0.03
	HF	4.80	6.70	10.47	10.43					

^1^ IVDMD_48_, in vitro dry matter disappearance at 48 h; GP_48_, cumulative gas yield at 48 h; *A*, the ideal maximum gas production; *B*, the sharpness of the gas production curve; *C*, the time at which half of *A* is reached; AGPR, the gas production speed when the gas production is 1/2 of the maximum. SEM, standard error of the difference of the means, *n* = 7. ^2^ S, substrate effect of CS supplementation level; I, interaction effect between substrate and CS supplementation level; L, linear effect of CS supplementation level; Q, quadratic effect of CS supplementation level.

**Table 3:** Effects of different substrates and CS supplementation level on rumen fermentation characteristics.

Item ^1^		CS Supplementation Level (mg/g Based on Substrate Weight)				SEM	*p*-Value ^2^			
		0	20	40	60		S	I	L	Q
pH	LF	6.20	6.14	6.13	6.16	0.045	<0.01	0.08	0.18	0.11
	HF	6.54	6.41	6.40	6.43					
Ammonia N, mg/dL	LF	44.5	42.2	37.9	34.6	0.58	0.01	0.680	<0.01	0.395
	HF	42.1	40.6	36.7	34.1					
MCP, mg/mL	LF	0.49	0.57	0.74	0.81	0.017	<0.01	<0.01	<0.01	0.395
	HF	0.45	0.56	0.64	0.67					
Total VFA, mmol/L	LF	101.2	113.6	126.1	128.8	12.03	<0.01	0.179	<0.01	0.079
	HF	93.1	96.7	111.7	112.5					
VFA patterns, % molar										
Acetate	LF	55.9	53.4	51.9	50.0	2.97	<0.01	0.342	<0.01	0.079
	HF	60.1	54.7	52.9 b	51.3					
Propionate	LF	16.2	17.5	19.0	20.0	0.39	0.041	0.035	<0.01	0.019
	HF	15.5	17.2	18.2	19.8					
Butyrate	LF	2.54	2.43	2.52	2.51	0.085	0.914	0.248	0.714	0.395
	HF	2.52	2.56	2.40	2.51					
A:P	LF	3.97	3.06	2.72	2.52	0.072	0.022	0.107	<0.01	<0.01
	HF	3.90	3.34	2.91	2.60					

^1^ VFA, volatile fatty acids; MCP, microbial crude protein; A:P, the ratio of acetate to propionate. ^2^ S, substrate effect of CS supplementation level; I, interaction effect between substrate and CS supplementation level; L, linear effect of CS supplementation level; Q, quadratic effect of CS supplementation level. SEM, standard error of the difference of the means, *n* = 7.

**Table 4:** Effects of substrates and increasing CS supplementation level on antioxidant capacity of fermentation system.

Item ^1^		CS Supplementation Level (mg/g Based on Substrate Weight)				SEM	*p*-Value ^2^			
		0	20	40	60		S	I	L	Q
SOD, U/mL	LF	93.9	99.3	99.7	104.8	1.31	<0.01	0.39	<0.01	0.62
	HF	90.7	94.2	95.4	96.5					
MDA, nmoL/mL	LF	1.63 a	1.53	1.49	1.39	0.035	<0.01	0.39	<0.01	0.54
	HF	1.82	1.73	1.63	1.63					
CAT, U/mL	LF	10.63	10.66	11.07	11.14	0.139	0.35	<0.01	<0.01	0.58
	HF	9.98	11.00	11.14	11.83					
GSH-Px, U/mL	LF	1095	1159	1178	1201	20.5	<0.01	<0.01	<0.01	0.44
	HF	988	992	1124	1232					
T-AOC, U/mL	LF	5.43	5.82	6.02	6.20	0.129	<0.01	<0.01	<0.01	0.29
	HF	4.78	5.03	5.32	6.35					
GSH, U/mL	LF	3.79	4.09	4.25	4.23	0.135	0.83	<0.01	<0.01	0.08
	HF	3.51	3.74	3.76	5.27					
GR, U/mL	LF	7.93	8.14	8.17	8.27	0.126	<0.01	0.04	0.032	0.06
	HF	7.32	7.53	8.24	7.57					

^1^ SOD, superoxide dismutase; MDA, malondialdehyde; CAT, catalase; GSH-Px, glutathione peroxidase; T-AOC, total antioxidant capacity; GSH, glutathione; GR, glutathione reductase. ^2^ S, substrate effect of CS supplementation level; I, interaction effect between substrate and CS supplementation level; L, linear effect of CS supplementation level; Q, quadratic effect of CS supplementation level. SEM, standard error of the difference of the means, *n* = 7.

**Table 5:** Effect of substrates and increasing CS supplementation level on ruminal bacteria alpha diversity based on OTUs.

Item		CS Supplementation Level (mg/g Based on Substrate Weight)				SEM	*p*-Value ^1^			
		0	20	40	60		S	I	L	Q
Coverage	LF	0.99	0.99	0.99	0.99	<0.01	0.715	0.089	0.289	0.793
	HF	0.99	0.99	0.99	0.99					
Chao	LF		1316	1314	1208	17.7	<0.01	0.951	0.508	0.064
	HF	1193	1203	1240	1237					
Ace	LF	1287	1285	1281	1203	16.1	<0.01	0.759	0.358	0.094
	HF	1179	1190	1213	1200					
Simpson	LF	0.020	0.024	0.027	0.038	<0.01	<0.01	0.139	0.292	0.903
	HF	0.035	0.044	0.035	0.032					
Shannon	LF	5.00	4.89	4.87	4.70	0.056	<0.01	0.157	0.278	0.953
	HF	4.71	4.65	4.75	4.77					
Sobs	LF	1031	1019	1029	968	11.1	<0.01	0.557	0.357	0.136
	HF	968	967	995	980					

^1^ S, substrate effect of CS supplementation level; I, interaction effect between substrate and CS supplementation level; L, linear effect of CS supplementation level; Q, quadratic effect of CS supplementation level. SEM, standard error of the difference of the means, *n* = 6.

**Table 6:** Microbial community analysis at the genus level (relative abundance > 1%) of microbiomes with increasing CS supplementation level in HF group.

Item, %	CS Supplementation Level (mg/g Based on Substrate Weight)				SEM	*p*-Value ^1^	
	0	20	40	60		L	Q
*Succiniclasticum*	18.9	21.2	20.4	19.1	3.066	0.982	0.564
*Bifidobacterium*	8.13	6.79	6.29	7.21	0.679	0.296	0.113
*norank_f_Muribaculaceae*	7.86	6.36	6.77	6.36	0.716	0.160	0.727
*Rikenellaceae_RC9_gut*	7.23	6.17	7.03	7.16	0.982	0.886	0.554
*Christensenellaceae_R-7*	5.59	5.37	5.12	5.04	0.479	0.384	0.897
*Lachnospiraceae_NK3A20*	5.95	4.85	4.78	4.95	0.420	0.119	0.148
*Prevotella*	2.35	3.92	3.17	5.09	0.699	0.027	0.810
*NK4A214_group*	3.07	2.71	2.72	2.52	0.199	0.080	0.664
*norank_f_F082*	2.47	2.66	2.98	3.20	0.287	0.028	0.373
*Desulfovibrio*	2.40	2.85	2.52	2.04	0.209	0.146	0.038
*norank_f_Eubacterium_coprostanoligenes*	2.61	2.73	2.21	1.96	0.200	0.013	0.366
*Family_XIII_AD3011*	2.47	2.52	1.98	1.75	0.242	0.022	0.559
*Ruminococcus*	1.74	2.32	2.38	2.13	0.187	0.154	0.039
*Fretibacterium*	1.38	1.36	1.84	2.03	0.312	0.098	0.733
*norank_o_WCHB1-41*	1.77	1.19	1.93	1.65	0.541	0.873	0.780
*Prevotellaceae_UCG-001*	0.64	1.82	1.36	2.42	0.486	0.037	0.899
*Eubacterium_nodatum*	1.61	1.45	1.62	1.50	0.199	0.865	0.921
*Pyramidobacter*	1.10	1.14	1.33	1.74	0.250	0.075	0.462
*Veillonellaceae_UCG-001*	0.99	1.18	1.32	1.23	0.231	0.427	0.577

^1^ L, linear effect of CS supplementation level; Q, quadratic effect of CS supplementation level. SEM, standard error of the difference of the means, *n* = 6.

**Table 7:** Microbial community analysis at the genus level (relative abundance > 1%) of microbiomes with increasing CS supplementation level in LF group.

Item ^1^	CS Supplementation Level (mg/g Based on Substrate Weight)				SEM	*p*-Value ^1^	
	0	20	40	60		L	Q
*Rikenellaceae_RC9_gut*	12.9	13.5	12.2	12.4	1.041	0.322	0.127
*Succiniclasticum*	12.4	13.8	12.3	9.16	2.912	0.402	0.439
*Bifidobacterium*	4.90	6.51	6.66	14.16	1.132	<0.01	0.017
*Muribaculaceae*	5.87	5.85	7.55	8.33	1.741	0.262	0.797
*Prevotella*	5.87	6.00	6.58	7.51	1.594	<0.01	0.066
*Lachnospiraceae_NK3A20*	5.06	4.27	4.11	6.49	0.666	0.181	0.028
*Christensenellaceae_R-7*	6.24	5.01	4.54	3.71	0.448	<0.01	0.667
*norank_f_F082*	2.88	2.90	3.66	5.61	0.348	<0.01	0.012
*Prevotellaceae_UCG-001*	2.81	4.63	4.06	4.35	0.891	<0.01	0.089
*Fretibacterium*	2.87	3.70	3.46	3.03	1.025	0.961	0.548
*NK4A214_group*	2.40	1.97	1.95	2.45	0.208	0.889	0.035
*Desulfovibrio*	3.19	2.19	1.85	1.21	0.221	<0.01	0.431
*norank_f_Eubacterium_coprostanoligenes*	2.03	1.94	1.77	2.45	0.208	0.255	0.082
*Ruminococcus*	2.46	1.92	2.02	1.13	0.247	<0.01	0.485
*Family_XIII_AD3011*	2.20	1.97	1.52	1.18	0.271	<0.01	0.844
*Pyramidobacter*	1.03	1.15	1.53	1.16	0.342	0.623	0.482
*VeillonellaceaeUCG001*	0.99	1.44	1.24	1.09	0.367	0.956	0.426
*Prevotellaceae_UCG-003*	1.14	0.91	1.13	0.75	0.278	0.445	0.779
*Sharpea*	0.59	0.83	0.76	1.71	0.150	<0.01	0.028

^1^ L, linear effect of CS supplementation level; Q, quadratic effect of CS supplementation level. SEM, standard error of the difference of the means, *n* = 6.

## Data Availability

Not applicable.
